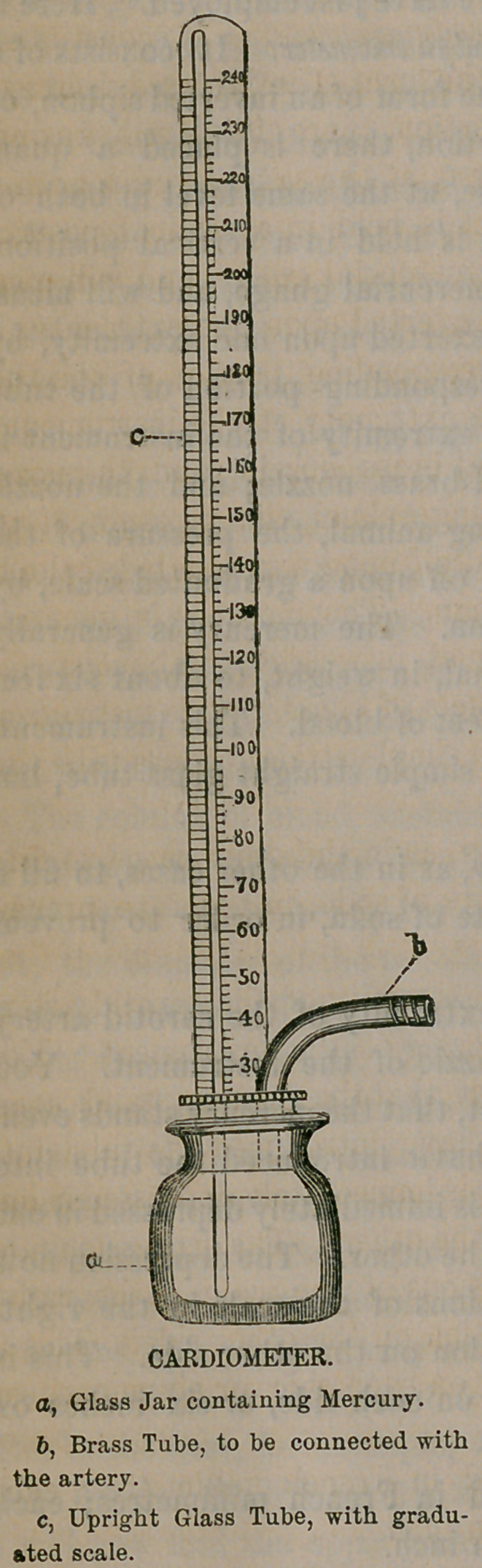# The Physiology of the Circulation

**Published:** 1860-05

**Authors:** John C. Dalton

**Affiliations:** Professor of Physiology and Microscopic Anatomy


### NEW YORK MONTHLY REVIEW

AND

### BUFFALO MEDICAL JOURNAL.

VOL. 15. MAY, 1860. NO. 12.

### ORIGINAL COMMUNICATIONS.

*The Physiology of the Circulation. A Course of Lectures delivered at the College of Physicians and Surgeons, New York, in the Fall Term of* 1859. By John C. Dalton, Jr., M.D., Professor of Physiology and Microscopic Anatomy.

### LECTURE V.

(September 27.)

The Arteries and Arterial Circulation—Elasticity of the Arteries—Action of Aortic Valves—Arterial Pulse—Mode of its Production—Its Retardation at a Distance from Heart—Experiments of M. Marey—Pressure of Blood in the Arteries—Experiment—Different Instruments for Measuring Pressure—Constant or Arterial Pressure—Oscillations from Action of Heart—From Movements of Respiration—Disturbance of Arterial Pressure from Various Causes—Struggling—Congestion—Ligature of Arteries—Hæmorrhage—Obstruction of Respiration—Experiment—Rapidity of Pulse—Action of Woorara—Opening of Chest—Experiment.

We come now, gentlemen, to the consideration of the second great division of the circulatory apparatus, namely—the arteries. I shall not occupy your attention with any detailed description of the anatomical structure of these vessels. I will simply remind you that they constitute a system of ramifying vascular tubes, everywhere continuous with each other, and communicating freely with the heart at one extremity, and with the capillaries at the other. These simple anatomical relations are the most important of those which it is necessary to recollect in studying the peculiar phenomena of the arterial circulation.

The first and most essential physical property of the arterial tubes is their elasticity. This property, as you know, is due mostly to the structure of their middle coat, which, in all the larger and medium-sized arteries, contains an abundance of elastic fibres, running both longitudinally and transversely, and arranged in successive layers. But in the largest arteries, these fibres encroach upon the internal and external tunics, to such a degree, that the whole vessel seems composed essentially of elastic tissue.

The characters which I have just mentioned can be seen very distinctly in this aorta of the ox. You have here a tube with very thick walls, distensible and elastic in every direction. It is precisely like a large tube of india rubber. You can stretch it longitudinally, or dilate it transversely, by forcibly drawing apart its walls; and it always returns upon itself, and instantly resumes its former shape.

You can appreciate, therefore, the effect which must be produced in such a series of ramifying tubes by the force of the cardiac impulse and the reaction of their elastic walls upon the circulating blood.

In the first place, the contraction of the heart produces a sudden distention of the entire arterial system, by driving into it the blood from the left ventricle. The contraction of the heart, however, being instantaneous, is immediately followed by a relaxation, and the arteries, by virtue of their elasticity, then react upon the blood, and compress it with a force nearly equal to that by which they were themselves dilated. Now, what becomes of the blood subjected to this reaction? It would, as you know, regurgitate into the cavity of the ventricle, were it not for the existence of the aortic valves.

I will now call your attention, briefly, to the peculiar arrangement of the aortic valves, and their mode of operation.

These valves consist, you remember, of a set of festooned membranous curtains, fibrous in structure, and capable of great resistance. They stand as a barrier between the cavity of the aorta and that of the ventricle, and bear the pressure of the blood under the reaction of the arterial system. In this aorta of the ox, the valves have been left entire at its cardiac extremity. By filling the vessel with water, from above downward, the valves, you observe, are closed, and the aorta remains full. If we now insert the nozzle of a syringe into its upper extremity, and forcibly inject it in a backward direction, the artery is distended at each stroke of the piston, and then reacts upon the contained fluid, just as it would react upon the blood in the natural state of the circulation. Wherever an artery can be felt by the touch, you easily perceive this alternate distention and reaction of the vessel. It is this distention of the arteries by the cardiac impulse, followed by their reaction, which is known as the phenomenon of the *arterial pulse.*

This arterial pulsation, which, as a fact, has been familiar to physiologists from time immemorial, was for a long period altogether misunderstood.

Singular as it may seem, the pulsation of the arteries in the earlier ages of medicine was thought to be a phenomenon of the same character as the expansion and collapse of the chest in respiration. Down to the time of Galen, the arteries were supposed to be filled with air, and their pulsation was supposed to be a kind of fanning or refrigerating process, by which the air was distributed to all parts of the body, so that the organs might be cooled by this bellows-like motion of the arterial tubes. Now, however, we know the fact to be quite different. The arteries are filled with blood, and their movement is caused by the alternate action of the heart and their own elastic resilience.

The pulsation itself, therefore, we understand, but we do not yet understand all its peculiarities and modifications. How is it, for example, that the pulse is not synchronous or simultaneous, in its occurrence, all over the body? We have one pulse at the heart, produced by the contraction of the left ventricle; another at the carotid, which some observers can distinguish as coming a little later than that at the heart; another at the wrist, which is very perceptibly later; and another at the ankle, in the posterior tibial, which is later still. This difference in the time of the arterial pulsations has been explained by supposing that the cardiac impulse travels outward like a wave, and so requires a certain time to reach the distant points of the circulatory system. But this explanation, though apparently so simple, is in reality insufficient. For we must recollect, that though the arteries are yielding and distensible, the blood itself is incompressible and inelastic. It must, therefore, transmit a mechanical impulse instantaneously in all directions, and cannot permit any delay in communicating the pulsations of the heart.

It has recently been found by accurate examination, that this is really the case. M. Marey has shown in a very satisfactory manner that the cardiac impulse is communicated, at one and the same instant, to the entire arterial system. All the arteries are distended simultaneously, by each stroke of the heart. The only difference in the pulsations near the heart, and at a distance, consists in the sudden or gradual manner in which the arterial distention takes place.

This fact is shown in the following manner: A long elastic tube is taken, and hung in several festoons between hooks which support it in its place. One end is connected with a forcing-pump, which injects through it, by alternate strokes, a stream of water, which escapes by the opposite extremity. Upon each festoon of the tube rests a little movable index, so arranged that it can be raised by a very slight force. At every stroke of the forcing-pump, therefore, the tube is distended, and each index is moved in a corresponding manner. Each index also marks upon a revolving cylinder of white paper in such a way as to register the rate and extent of its own movement. When the machine is in operation, accordingly, the different curves marked upon the paper show the exact manner in which different parts of the tube are distended by the strokes of the forcing-pump.

Now, it is found by such an experiment, that the expansion of the tube, in different parts of its length, *begins* at the same instant. The only difference is in the extent of the expansion, and the rapidity with which it is accomplished.

In the end of the tube nearest the forcing-pump, as in the arteries nearest the heart, the expansion is very wide and very sudden. The tube is almost instantly dilated to its utmost extent, while the reaction which follows is slow, and occupies all the rest of the time of a single pulsation. At a little distance further on, the expansion is more gradual, taking a longer time to reach its maximum, and is also less in extent than before. This is because, in the immediate neighborhood of the heart, almost the whole force of the cardiac impulse is occupied in expanding the vessel; and this expansion is, therefore, quick and sudden, like the stroke of the heart itself. But after that, comes the elastic reaction of the aorta, which urges the blood onward into the next portion of the arterial tube. Here, accordingly, the distention of the vessel is due partly to the stroke of the heart, which is quick, and partly to the resiliency of the aorta, which is slow. Its expansion is *completed*, therefore, a little later than in the former instance, though it *begins* at the same instant. For a similar reason, the expansion is less in extent at a distance from the heart, because its impulse is at first partly expended in dilating the aorta behind the point examined, and is afterwards replaced by the reaction of the same vessel.

In the neighborhood of the heart, accordingly, the arteries expand with a brusque and violent motion, but their reaction is slow and gradual. This is followed by another brusque and sudden dilatation; and so on again. At a greater distance, the dilatation becomes more gradual, and more proportionate to the reaction; while at a greater distance still, the expansion of the artery occupies an equal time with its reaction; so that the expansion and reaction of the vessels become more uniform, similar, and equal to each other, the farther we recede from the centre of the circulation.

This explains the apparent retardation of the pulse towards the extremities of the arterial system. Near the heart, the pulsation of the arteries appears to be synchronous with the cardiac impulse, because their distention is so rapid and sudden; but at a distance, it seems to be later, because it takes a longer time for its completion.

You see, then, how the whole movement of the blood is equalized by the elastic reaction of the arteries; and that, though the blood is thrown out from the left ventricle in distinct and interrupted jets, its motion afterwards becomes constantly more steady and uniform. This change is owing entirely to the elasticity of the vessels. As the blood is discharged from the heart and enters the aorta, it is at once subjected to the reaction of the entire arterial system, and is forced to circulate under this pressure.

This brings us to the study of another very important point in the history of the circulation, viz., the *arterial pressure.*

As soon as the blood has left the heart and is contained in the arterial system, it is subject to a very remarkable, firm, and constant compression, which is exerted upon it by the elastic walls of the arteries themselves. You remember, however, that the arteries consist of a series of cylindrical tubes, whose cavities are continuous with each other throughout; and this reaction, therefore, which they exert by their elasticity, is equally felt throughout the entire system.

Now, the pressure which is thus exerted upon the blood in the arterial system can be shown by a very simple experiment.

I have here a dog which I will etherize, and then insert the extremity of a narrow glass tube into the carotid artery. You will then see the blood forced upward into the tube in such a way as to indicate the extent and power of the arterial compression. The carotid is first to be exposed and separated from the pneumogastric nerve and other adjacent parts. You now see the vessel running along the bottom of the wound, and pulsating with its usual force. Its upper portion is then to be tied, and the vessel being temporarily secured by the fingers, an opening is made into it at the point where the glass tube is to be applied. The lower end of the instrument is furnished with a short piece of india-rubber tubing, terminated by a brass nozzle, which is inserted into the artery and secured by ligature. The lower part of the tube is filled with a solution of carbonate of soda, to prevent the too rapid coagulation of the blood.

Now, on releasing the artery and allowing the blood to pass into the glass tube, it rises; you observe, nearly to the top of the tube, to a height of about five feet and a half. Sometimes the pressure of the blood in the carotid of the dog is equal to five feet; sometimes five feet and a half, and sometimes six feet. This difference depends not so much upon the size of the animal as upon the condition of the circulatory system at the time.

You see, moreover, that the column of blood is not quite steady in the tube, but that it sometimes rises near the upper end, and then, in a few seconds, falls several inches below it. This motion corresponds with the movements of respiration. During inspiration the column of blood is lower, and during expiration it is higher than usual. Beside this slow oscillation, there is also another rapid one. The level of the blood moves up and down by a series of short and tremulous oscillations, which are synchronous with the pulsations of the heart. Both these oscillatory movements we shall study more fully hereafter. But notwithstanding them, you see that the level of the blood is constantly maintained in the tube, at or above a certain height, by a continuous and lasting pressure.

The column of blood, sustained in this way, is very nearly the same in animals of different size. There is but little difference in this respect between the horse, the dog, and so small an animal as the rabbit; the diameter of the tube employed being, of course, proportionate in each instance. There is also but little difference in the different arteries of the same animal. The column of blood stands at nearly the same level, whether the tube be inserted into the carotid, the femoral, or one of the smaller branches of these vessels; for the arteries all communicate with the aorta, and of course the pressure is transmitted almost equally to every point.

Where the arteries inosculate freely with each other by transverse branches, this pressure may even be shown by inserting the tube into a vessel which is separated from the heart by division or ligature. This is the case, for example, with the carotids. In this animal, we have divided the left carotid and secured both extremities by ligature. I will now find the upper extremity of the vessel, and, taking off the ligature which was placed upon it, introduce into it the brass nozzle of our instrument, as before. You see, the blood passes again into the tube from the upper portion of the carotid, and though the column of blood is not so high as before, it is, nevertheless, a very considerable one, and shows the communication of the arterial pressure from the other vessels of the head and neck.

There are various instruments which have been contrived for showing the pressure of the blood in the vessels, more compact and convenient for use than the long glass tube which we have just employed. Here is one of them, which is called the *Hæmodynamometer.* It consists of a bent glass tube, open at both ends, in the form of an inverted siphon, or letter U. In the lower curved portion, there is placed a quantity of mercury, which stands, of course, at the same level in both of the upright limbs, when the instrument is held in a vertical position. It is calculated to act, therefore, as a mercurial guage, and will measure accurately any excess of pressure exerted upon one extremity, by the descent of the mercury in the corresponding portion of the tube and its rise in the opposite limb. One extremity of the instrument is furnished with a short flexible tube and brass nozzle; and the nozzle being inserted into the artery of a living animal, the pressure of the blood, and its oscillations, may be read off upon a graduated scale, by the rise and fall of the mercurial column. The mercury is generally displaced about six inches, which is equal, in weight, to about six feet of water, or a little over five and a half feet of blood. This instrument, therefore, gives the same result as the simple straight glass tube, but in a more compact form.

Before using this tube, it is necessary, as in the other cases, to fill a portion of it with a solution of carbonate of soda, in order to prevent the coagulation of the blood.

I will now take up again the lower extremity of the carotid artery in this animal, and insert into it the nozzle of the instrument. You observe, before placing the two in contact, that the mercury stands evenly and at zero in both limbs. Now I have introduced the tube into the artery, and you see that the mercury is immediately depressed in one branch of the tube, and that it rises in the other. The depression now varies between sixty and sixty-five divisions of the scale in the right-hand tube, and there is a similar elevation on the other side. This is nearly equal to three inches of mercury on each side, or six inches on both sides.

The divisions of the scale are marked in French millimetres; each millimetre being equal to about $\frac{1}{25}$ of an inch.

Now notice, gentlemen, if you please, the different effects of the arterial pressure and the cardiac pressure. The pressure of the arterial system is indicated by the entire and constant displacement of the mercury in the limbs of the instrument; while the effect of the cardiac pressure is the incessant oscillation which you observe in the level of the mercury. The liquid is depressed more strongly as the heart contracts, and less so as it relaxes. The column now varies between sixty and seventy millimetres on each side. That is, the force of the heart's contraction is equal to ten millimetres on each side, or twenty millimetres in the whole; which is rather less than one inch of mercury, or one foot of blood.

CARDIOMETER.

*a*, Glass Jar containing Mercury.

*b*, Brass Tube, to be connected with the artery.

*c*, Upright Glass Tube, with graduated scale.

The excess of the cardiac pressure, therefore, is nearly equal to one foot of blood, while the reaction of the entire arterial system is between five and six feet.

Here is another instrument, constructed upon the same principle with the other, but still more compact in form, and more convenient. It is called the *Cardiometer*. It consists of a small, but strong, wide-mouthed glass bottle, with a closely-fitting brass stopper. Through the stopper there passes a short brass tube, which is bent at right angles, and which is furnished with a nozzle, to be inserted into the artery. From the lower part of the bottle there also rises a narrow glass tube, about ten inches in height, open at both ends. The bottle is filled with mercury, and when the brass nozzle is made to communicate with an artery, the pressure of the blood acts upon the mercury and forces it up into the glass tube to a corresponding height. This instrument is found to be much better than the other for showing rapid variations of pressure by the oscillations of the mercury.

To illustrate its operation, I will detail several experiments which I ave performed with it in the following manner:

*Experiment.*—A healthy dog, weighing a little over 31 pounds, was etherized six hours after feeding, and the cardiometer applied to the cardiac extremity of the left carotid artery.

At first, the mercury oscillated in the upright tube as follows:

130 to 160 (millimetres.)
135 to 165.
150 to 170.

These oscillations were mostly due to the effect of the respiratory movements, the mercury going up to 160, 165, and 170 at the time of expiration, and falling to 130, 135, and 150 at the time of inspiration. The *cardiac impulses*, of which there were two or three for each inspiration and each expiration, were not more than five millimetres each; so that the mercury mounted, say from 130 to 160 at one expiration, showing, during its ascension, several small cardiac oscillations, of five millimetres each.

After the animal was more thoroughly etherized, so that the respirations became very calm, the disturbing influence of the respiratory movements disappeared, and each cardiac impulse was marked by an oscillation of 10, 12, or even 15, as follows:

160 to 175.
190 to 200.
150 to 162.

Cardiac pulsations=10 to 15 millimetres.

A few minutes afterward, the animal still remaining calm, but beginning to recover a little from the effects of etherization, the mercurial oscillations again indicated slightly the disturbing influence of the respiratory movements, as follows:

145 to 152.
140 to 150.

Cardiac pulsations=7 to 10.

Still later, the movements of respiration created an oscillation of 10 millimetres, as follows:

140 to 150.

The cardiac pulsations being only 5 each.

The animal was then freshly etherized, when the respiratory oscillations again disappeared, and the cardiac pulsations at the same time became more strongly marked, as follows:

150 to 160.
165 to 180.
150 to 165.

Cardiac pulsations=10 to 15.

One grain of woorara in solution was then injected into the subcutaneous areolar tissue of the left thigh. The poison, in this instance, was unusually long in producing its effects, (viz., from ten to fifteen minutes,) owing, probably, to the etherized condition of the animal. During this interval, the following phenomena were observed:

Soon after the injection, the cardiac pulsations were well marked, equalling 10 millimetres each; the respiratory oscillations at the same time being only 5 millimetres, as follows:

150 to 160.
155 to 165.
160 to 170.

Cardiac pulsations=10.

But during the access of laborious respiration, which came on once or twice, the *respiratory oscillations* became very extensive, equalling 110 millimetres, thus:

110 to 220.

As soon as the poison began to produce its specific effects, the trachea was opened, the nozzle of a bellows inserted into it, and artificial respiration kept up. The *cardiac pulsations* were then pretty constant, at five millimetres each. But the effect of the artificial insufflation upon the *respiratory oscillations* was just the opposite of that produced by natural inspiration.

In natural respiration, the mercury rises at the time of expiration, and falls at the time of inspiration. Now, however, as might be expected, the mechanism of respiration being changed, its effect upon the arterial pressure was different. At each artificial insufflation the mercury mounted in the tube for 20 or 25 millimetres, as follows:

155 to 180, and descended again, in the intervals, to its former level; the constant or arterial pressure remaining about the same, viz., 150 or 155 millimetres.

When the artificial insufflation was suspended the arterial pressure was at once increased, the mercury mounted in the tube to 200 or thereabout, and oscillated at that level by cardiac pulsations of 10 millimetres each, thus:

190 to 200;
200 to 210; but immediately fell again to 155, when the insufflation was recommenced, the cardiac pulsations again becoming reduced to five millimetres.

When the animal had become fully affected by the woorara, so that there was complete loss of consciousness, no voluntary movement, and no respiratory movement of the lips or nostrils, the oscillations produced by insufflation were as follows:

150 to 180.

Cardiac pulsations=5.

The chest of the animal was then opened, and the heart and the great vessels exposed to view. A little blood was lost in the operation. Immediately afterward, the cardiac impulses were sensibly weakened, being reduced to three millimetres each; but in a short time they recovered strength a little, and were again five millimetres. Owing to the removal of the thoracic parietes, also, the effects of *insufflation* on the mercurial oscillations now disappeared, and the only oscillations perceptible were those due to the cardiac movements.

Very soon after opening the chest the entire arterial pressure diminished very considerably, the level of the mercury falling 100 millimetres, and its oscillations being as follows:

45 to 50.
50 to 55.
70 to 75.

Cardiac pulsations=5.

On suspending insufflation, the pressure in the arteries was increased as before, and the cardiac pulsations became very long and distinct, thus:

100 to 150.
130 to 200.

Cardiac pulsations=50 to 70.

On recommencing insufflation, the oscillations again fell to their former level, as follows:

50 to 55.

Cardiac pulsations=5.

The experiment was then terminated.

From these observations, gentlemen, it is easy to see that there are three principal elements in the pressure or force with which the blood circulates in the arterial system.

The first of these is the *constant* or *arterial* pressure. This depends upon the reaction exerted by the entire arterial system by means of its elastic parietes. The blood, contained in a series of branching tubes provided with elastic walls, and completely filling their cavities, is subjected thereby to a steady and continuous pressure. This pressure is felt equally, or nearly so, at all parts of the arterial system, and in all directions, as if an elastic bag were filled with a fluid which had been forcibly injected into it. Accordingly, if an opening be made in any part of the arterial system, the blood is driven out with a certain force by the reaction of the vessels themselves. If a tube filled with mercury be inserted into the mouth of a divided artery, and secured by ligature, the blood will displace the mercury from its level, until the weight of the mercurial column exactly counterbalances the reaction of the arteries. The arterial pressure can thus be measured; and we have found that it is usually equal to about 150 millimetres, or six inches of mercury.

Beside the constant or arterial pressure, however, the blood is also subject to an intermitting action, viz., that of *the cardiac impulse.* At every contraction of the heart, more blood is forcibly thrown into the arteries, and, of course, the pressure is momentarily increased. When the heart relaxes, the superabundant portion of blood passing steadily away by the capillaries and veins, the increased pressure is taken off, and the arterial reaction only remains. There is, accordingly, a series of rapid oscillations in the column of mercury, corresponding with the action of the heart. At every beat the level of the mercury rises, at every relaxation it falls. The extent of these oscillations measures the force of the heart and the resistance of the artery. If the arteries were entirely unyielding, the whole force of the heart's contraction would be manifested by the rise of the mercury in the tube of the cardiometer. But the arteries are distensible and elastic; so that a portion of the heart's impulse is occupied in dilating their walls, and only a part of its force is shown by the immediate rise of the mercurial column. We have, therefore, a constant pressure, due to the arterial elasticity, and an oscillating pressure, due to the superior force of the heart's pulsations.

While the arterial pressure, also, is the same in all parts of the body, the influence of the cardiac pulsations diminishes from the heart outward. For at a distance from the heart, in the radial or tibial artery, for example, the force of the heart's impulse has already been divided and subdivided in distending the arterial coats; and the elastic vessels, also, return to the blood, in the intervals of pulsation, more and more of the force with which they were distended; so that the difference in the pressure on the blood, corresponding with the heart's pulsations, becomes less and less, from the centre to the circumference; but the mean or constant pressure remains the same.

The third force exerted upon the blood in the arteries is that of the *respiratory movements.* The effect of this is seen in the rise and fall of the mercury every time that the chest expands and collapses. At the moment of expansion, the pressure is lifted off from the heart and large vessels by the rising of the walls of the chest; the tension of the arterial system is consequently diminished, and the mercury falls a little in the cardiometer. When the chest collapses again, on the other hand, this pressure is restored, and the mercury rises to its former level. There results, accordingly, a series of oscillations, which are distinct from those produced by the cardiac pulsations, and which are synchronous with the movements of respiration. These respiratory oscillations are but slightly perceptible in the ordinary condition of the animal, and not at all so when the breathing is very easy and quiet; for then, the inspiration is so slow and gradual, that the air readily penetrates the lungs, and immediately counterbalances the diminished pressure of the thoracic parietes. But whenever the breathing becomes rapid and laborious, the difference of pressure in inspiration and expiration is so marked, that it produces a sensible effect on the rise and fall of the mercurial column.

There are, accordingly, as we have seen in various experiments, two sets of oscillations in the cardiometer—one of them more rapid, corresponding with the arterial pulse—the other a slow one, corresponding with the movements of respiration. As each movement of respiration, also, corresponds in time with several successive cardiac pulsations, the movements of the mercurial column bear the same relation to each other. At each expiration the mercury rises in the tube, by three or four short successive strokes, to its full height, and then falls, during inspiration, by a descending series, to its former level. These appearances have been mistaken by some experimenters, and have been supposed to indicate a peculiar irregularity in the force of the heart's action; but a little observation will show that they are due to the cause I have just described; for the ascending movements of the mercurial column always take place at the time of expiration, and the descending movements at the time of inspiration.

These respiratory oscillations sometimes reach the extent of thirty millimetres or upward, while the cardiac impulses are not more than five or ten millimetres each. But when the respiration again becomes quiet, the disturbing influence of its movements disappears, and the only oscillations then perceptible are those due to the cardiac pulsations.

Before leaving this part of the subject, gentlemen, I will call your attention to some circumstances which modify the pressure of the blood in the arterial system.

1st. One of these circumstances is the condition of rest or activity of the animal subjected to experiment.

While the animal is in a quiescent condition, the arterial pressure is moderate—averaging about 150 millimetres; but as soon as he begins to make any exertion, it increases, and may reach 175 or 200 millimetres. If you were to wake up this animal from his etherized condition and make him struggle or cry, you would immediately see the arterial pressure very much increased. This is because, in the act of struggling, the muscles of the trunk and extremities are contracted, and the chest is forcibly compressed. This unusual compression of the chest not only crowds the blood from the heart and large vessels into the arteries, and so increases the tension of these vessels, but also has the effect to engorge them by a backward action through the veins and capillaries. The arterial system is therefore fully distended, and its pressure upon the blood increased, during the act of struggling.

2nd. A similar increase of the arterial pressure is produced by whatever causes a general or local congestion of the arterial system. Thus, anything which limits or confines the space occupied by the arteries, without at the same time diminishing the quantity of blood contained in them, will produce an increase of the arterial pressure. Now the ligature of an artery has precisely this effect. If the femoral artery be tied, and the circulating fluid thus prevented from gaining access to the corresponding limb, all the blood of the body will at once be distributed to the remainder of the arterial system. The quantity of blood, therefore, will be increased in proportion to the space which it occupies, and a corresponding increase of tension will be manifested throughout the rest of the arterial system.

Bernard has found, for example, that on applying the cardiometer to the left carotid artery of a dog, the minimum pressure was 110 millimetres; but after tying the carotid of the opposite side, it rose to 165 millimetres. On another occasion, the pressure rose, owing to the same cause, from 150 to 185 millimetres. And the larger the number of arteries tied, the greater will be the excess of pressure produced in the rest of the circulatory system.

It is evident, accordingly, that if the cardiometer be applied in such a way as to cut off the access of the blood to any considerable part of the vascular system, it will disturb the circulation in the same way as if the vessel were simply ligatured at that point, and the instrument will indicate an excessive and unnatural pressure. In order to avoid this difficulty, the cardiometer should be applied to some part of the arterial system where it will not materially interfere with the passage of the blood to the parts beyond. For this purpose, the carotid arteries are by far the best in the body. They are easily exposed and of a convenient size, and their branches inosculate so freely, in the head and neck, with each other and with the vertebrals, that the blood still finds its way readily into these parts after one of the carotids has been obstructed by the instrument. But in most other regions of the body, the application of the cardiometer upon the main artery cuts off all the blood from the part, and produces an unnatural rise in the arterial pressure.

It is for this reason, more than any other, that the cardiometer gives different results when applied to arteries in different regions of the body. Milne Edwards, for example, in his admirable work on Comparative Anatomy and Physiology, mentions that the inequality of pressure in the various arteries is not proportional to their distance from the heart, nor the same in different regions of the body; and that the pressure of blood in the femoral arteries, for example, is greater than that in the carotids.

This is because, when the cardiometer is applied to the femoral artery, the arterial current, as I have already mentioned, is *blocked* or stagnated in the thigh; but when it is applied to the carotid, the current is left nearly free and natural, owing to the abundant arterial inosculation about the neck and head.

If the proportion of blood in the vascular system be diminished, on the other hand, the arterial pressure falls in a corresponding degree. The abstraction of blood by hæmorrhage produces this effect. If the bleeding be moderate in amount and rapidity, it affects both the arterial and the cardiac pressure. But the cardiac pulsations feel the effect of the hæmorrhage more quickly than the steady pressure due to the reaction of the arterial walls. The oscillations of the mercury, accordingly, are first diminished in extent, as the force of the heart's action is lessened; and afterward the arterial pressure is also reduced, owing to the diminished quantity of blood in the vascular system. Bernard once applied the cardiometer to the carotid artery of a dog, and then subjected the animal to a moderate bleeding from the jugular vein. During the first nine minutes of the bleeding, the oscillations of the mercurial column, due to the heart's pulsations, were diminished in extent from 35 millimetres to 25, 20, 15, 10, and 5. But the arterial pressure remained nearly steady at 110 millimetres, until the tenth minute, when it fell to 95, and afterward to 90, 85, and 80 millimetres.

It is noticed, also, that the arterial pressure recovers itself very rapidly after stopping the hæmorrhage, but the cardiac pulsations remain enfeebled for a considerable time longer. This is undoubtedly because the *mass* of the blood is very soon replaced after a hæmorrhage, by absorption of serous fluid from the tissues, and the physical distention of the arteries is immediately recovered; but the *chemical constitution* of the blood is less easily restored, and the heart continues to feel this change until it is again rectified by the process of nutrition.

3d. The arterial pressure is also very much increased by any temporary obstruction to the respiration. We already understand why it should be so, since we have seen, in a previous lecture, that the immediate effect of an obstruction to the breathing is a congestion of the arterial system. By means of the cardiometer we can demonstrate and measure the extent of this congestion. If the instrument be applied to the carotid artery, and the respiration of the animal be then arrested, the mercurial column immediately begins to rise in the tube to a higher level.

In the experiment which I related to you, for example, the level of the mercury rose at one time, after stopping respiration, from 150 to 200 millimetres, and oscillated about that point by pulsations of ten millimetres each. In another instance, the arterial pressure, which had fallen to 60 or 65 millimetres in consequence of the opening of the chest, rose to 150 and 200 after stoppage of respiration.

The heart's pulsations, also, are increased in extent after the breathing has been suspended for a short time, amounting sometimes to 125 or 130 millimetres. The level of the mercury is then thrown up at each pulsation to 250 or 260 millimetres, and sinks back in the intervals to 150 or 170. During the latter part of the process, however, when the congestion of the arterial system passes off and that of the heart begins, both the arterial pressure and the cardiac oscillations are again reduced, and the mercury gradually falls in the tube, with the decreasing force of the circulation.

The following experiment will illustrate the alterations which take place in the vascular pressure after stoppage of the respiration:

*Experiment.*—A dog was poisoned with woorara, and artificial perspiration kept up while the thoracic duct was exposed at the root of the neck, and a silver canula inserted into it, for the purpose of collecting the chyle. The chyle was collected in this way for half an hour, after which the canula was withdrawn from the duct.

The cardiometer was then applied to the left carotid artery. The artificial respiration was kept up by insufflations through a bellows inserted into the trachea. The insufflations were made at the rate of forty per minute, and were moderate in force. They exerted no perceptible effect upon the oscillations of the mercury, which were altogether synchronous with the movements of the heart. The movements of the mercurial column were at first as follows:

140 to 150.

135 to 145.

Cardiac pulsations=10.

After a short time the cardiac pulsations became feebler and more frequent, viz., 150 to 160 per minute, and as follows:

125 to 130.
130 to 135.

Cardiac pulsations=5.

The insufflations were then stopped. The effect of the stoppage was to raise the arterial pressure, and almost simultaneously, also, to increase the extent of the cardiac pulsations. The cardiac pulsations also became less frequent, and more easily counted. They were as follows:

| Oscillations of the mercury. | Cardiac pulsations. | Oscillations of the mercury. | Cardiac pulsations. |
|---|---|---|---|
| 120 to 130 | 10 | 150 to 260 | 110 |
| 120 " 140 | 20 | 150 " 250 | 100 |
| 90 " 150 | 60 | 150 " 245 | 95 |
| 70 " 170 | 100 | 140 " 240 | 100 |
| 110 " 200 | 90 | 130 " 240 | 110 |
| 130 " 260 | 130 | 150 " 250 | 100 |
| 140 " 240 | 100 | 140 " 240 | 100 |
| 80 " 170 | 90 | 140 " 240 | 100 |
| 100 " 210 | 110 | 140 " 255 | 115 |
| 150 " 240 | 90 | 140 " 240 | 100 |
| 100 " 210 | 110 | 150 " 250 | 100 |
| 140 " 270 | 130 | 140 " 255 | 115 |
| 150 " 270 | 120 | 140 " 260 | 120 |
| 140 " 240 | 100 | 140 " 260 | 120 |
| 130 " 240 | 110 | 130 " 250 | 120 |
| 120 " 220 | 100 | 120 " 230 | 110 |
| 160 " 260 | 100 | 110 " 230 | 120 |
| 170 " 270 | 100 | 90 " 195 | 105 |
| 130 " 200 | 70 | 80 " 185 | 105 |
| 120 " 220 | 100 | 90 " 210 | 120 |
| 150 " 240 | 90 | 90 " 220 | 130 |
| 170 " 270 | 100 | 85 " 210 | 135 |
| 140 " 220 | 80 | 80 " 200 | 120 |
| 120 " 230 | 110 | 90 " 215 | 125 |

| Oscillations of the mercury. | Cardiac pulsations. | Oscillations of the mercury. | Cardiac pulsations. |
|---|---|---|---|
| 80 to 200 | 120 | 90 to 135 | 45 |
| 70 " 195 | 125 | 80 " 130 | 50 |
| 90 " 210 | 120 | 70 " 110 | 40 |
| 80 " 210 | 130 | 65 " 80 | 15 |
| 80 " 210 | 130 | 60 " 70 | 10 |
| 90 " 220 | 130 | 55 " 65 | 10 |
| 100 " 190 | 90 | 50 " 60 | 10 |
| 90 " 165 | 75 | 50 " 55 | 5 |
| 90 " 180 | 90 | 40 " 45 | 5 |

From this point the oscillations continued to diminish in height and extent, until they became altogether imperceptible.

By the use of the cardiometer we can satisfy ourselves of many important points in regard to the effect of various conditions upon the heart's action on the one hand, and the arterial pressure on the other. I have already mentioned to you that almost universally a *rapid* pulse is deficient in strength, while a *slow* pulse acts with more force and vigor. This can readily be seen in the variations which show themselves while experimenting with the cardiometer. I have almost invariably found that whenever the pulse of the animal becomes accelerated during an experiment, the oscillations of the mercury diminish in extent; while, if the pulse becomes slower, the oscillations are sensibly increased, though the constant pressure may remain unaltered.

In one experiment, for example, while the pulse was 160 per minute, the mercurial oscillations were 10 millimetres each; but after the pulse had fallen to 130 per minute, the oscillations were 15 millimetres each. At another time, the pulse rose in frequency to 150 or 160 per minute, and the oscillations were at the same time reduced from 10 to 5 millimetres each. On one occasion the force of the heart's pulsations having been reduced to 3 millimetres each, the pulse rose at the same time to 190 per minute. The force of the cardiac pulsations, therefore, is in inverse ratio to their rapidity.

I have observed, also, that the ordinary varieties of *woorara*, which act so powerfully on the voluntary muscles, exert little or no specific influence on the action of the heart. It may even increase somewhat the force of the heart's movements, while it lessens their rapidity, by destroying the consciousness of the animal, and thus preventing his being excited or agitated by external causes. The operation of *opening the cavity of the chest*, on the contrary, immediately depresses considerably both the cardiac pulsations and the arterial pressure, the pulse being at the same time very much increased in frequency. We can never, therefore, inspect the action of the heart, in its perfectly normal condition, by opening the chest. After this operation, the movements of the organ are always much more rapid than natural, and enfeebled to a corresponding degree. For the removal of the thoracic parietes takes away the external support from the lungs and arch of the aorta, and so diminishes the tension of the whole arterial system; while the exposure of the heart, and its contact with air, produce a comparatively irritable and enfeebled state of its muscular walls.

These points are illustrated by the following experiment:

*Experiment.*—A full-grown, healthy dog, weighing about 20 pounds, was etherized in the early part of the day, and the left carotid artery exposed by dissection, and separated from the surrounding parts for about two inches of its length. The wound was then closed by a suture, and the animal left to himself.

At half past one, P. M., the dog had completely recovered from the effects of the ether. He suffered no apparent inconvenience from the wound in the neck. When placed upon the table, his pulse was 174 per minute, of good quality.

The animal was then held in position, by assistants, upon his back, where he remained perfectly quiet, and without struggling. The wound was then opened and the cardiometer applied, in the usual way, to the left carotid artery. This operation did not produce any visible agitation in the animal, nor any sign of pain.

Immediately afterward, at 15 minutes before two, the pulse was 160 per minute, and the oscillations of the mercury in the cardiometer as follows:

135 to 145.
140 " 150.
130 " 140.
125 " 135.

Cardiac pulsations=10.

The animal remained, during this time, perfectly quiet, with a calm and uniform respiration.

At five minutes before two o'clock, the pulse had fallen to 130 per minute, and the oscillations were as follows:

125 to 140.
120 to 135.
120 to 135.

Cardiac pulsations=15.

At four minutes before two, one grain of woorara, in solution, was injected under the skin of the abdomen. The only immediate effect of this operation was a slight increase in the rapidity of the pulse, together with a diminution in force, the oscillations being reduced to ten millimetres each. This was probably owing to the slight degree of pain inflicted by the injection.

At five minutes past two, the pulse was again reduced to 140 per minute.

Oscillations, 125 to 140. Cardiac pulsations=15.

At ten minutes past two, the signs of poisoning by woorara became evident. The trachea was opened, the nozzle of a bellows inserted into it, and artificial respiration kept up.

As soon as the disturbance consequent on the temporary obstruction to respiration had ceased, the pulse was found to have decidedly diminished in frequency and gained in force.

At fifteen minutes past two, the pulse was 62 per minute, and the oscillations were as follows:

125 to 150.
135 to 160.
125 to 150.

Cardiac pulsations=25.

At half past two the pulse was 80 per minute, and the oscillations

135 to 165.
130 to 160.

Cardiac pulsations=30.

At thirty-five minutes past two, the chest was opened in the usual manner, so as to expose the heart and lungs. Immediately afterward it was found the pulse was very much accelerated and excessively reduced in force. The arterial pressure was also much diminished. The pulse was 190 per minute, and the oscillations as follows:

90 to 93.
92 to 95.
87 to 90.

Cardiac pulsations=3.

On cutting away the pericardium and fully exposing the heart, it was seen that the action of the organ was not perceptibly weaker than it usually is, after being exposed to view in this way. The movements of the heart continued, under the use of the artificial respiration, as in other similar experiments; but the mercury in the cardiometer-tube sank gradually below 80, 70 and 60, and finally below 40 millimetres, the oscillations remaining very weak, and measuring only two or three millimetres each.

On stopping the artificial respiration, the mercury immediately rose, as usual, in the cardiometer, but only to 160 or 170 millimetres, and afterward sank out of sight again as the heart's action finally ceased.

I will now, gentlemen, terminate this lecture by stopping the respiration in this animal, to whose carotid artery we attached the mercurial guage with a double tube. For this purpose, I will inject into the femoral vein a small quantity of solution of woorara. The injection is made slowly, so as not to produce any mechanical disturbance of the circulation.

The displacement of the mercury, you observe, on each side of the instrument is between thirty and forty divisions of the scale, and is gradually diminishing. It is less than usual, owing to the long time the animal has been kept under the influence of ether. It is now about thirty-five divisions. The respiration, within a minute and a half after the injection of woorara, is already becoming very quiescent. You observe a twitching about the eyes of the animal, which can often be seen during the operation of this poison.

At this time, the pulsations of the heart continue with a tolerable degree of force and regularity. The mercury stands at twenty to twenty-five divisions. Now the respiration has entirely ceased, and I can distinctly feel the pulsations of the heart through the ribs. The mercury has fallen below twenty, and its oscillations are very weak.

Now, you observe, the mercury begins to rise again in the tube of the instrument. It is now twenty-five divisions, now twenty-seven, and now thirty. This increased arterial pressure, you will understand, is owing to the stoppage of respiration and the obstruction of the capillary circulation. The column of mercury has now risen to forty divisions. The pulsations of the heart at this time are feeble, but recur with the same rapidity and regularity as before. The mercury still has a tendency to rise, showing that the arterial pressure is very much greater than it was a moment after the stoppage of respiration. It has now reached forty-five, and now forty-seven and a half.

Now the arterial pressure begins suddenly to diminish. The mercury falls to forty-two, thirty-seven, thirty-five, thirty. The last stage of the process has now commenced. Regurgitation takes place from the aorta, and the heart becomes congested and paralyzed, while the distention of the arterial system begins to subside and disappear.

You see, now, the phenomenon which is always to be noticed at this stage, viz.—the mercury sinks toward the same level on both sides of the instrument. The pulsations of the heart are longer than before; they occupy a greater interval, and do not recur so rapidly. The mercury has subsided to twenty-five divisions. The heart's pulsations are still very readily felt through the walls of the chest, and are absolutely synchronous with the oscillations of the mercury in the instrument. At every contraction of the heart, the mercury is elevated in the tube; but at each relaxation, the mercury falls farther back than before, because the blood now regurgitates from the aorta into the heart.

The left auricle and ventricle, as well as the right cavities, are now beginning to be distended and paralyzed. The mercury has already fallen to twelve or thirteen divisions, and will very soon come to a level on both sides of the instrument, and finally stand at zero, as the circulation comes to an end.

## Figures and Tables

**Figure f1:**